# Desmoids in familial adenomatous polyposis are monoclonal proliferations

**DOI:** 10.1054/bjoc.1999.1007

**Published:** 2000-02

**Authors:** S B Middleton, I M Frayling, R K S Phillips

**Affiliations:** The Polyposis Registry, Imperial Cancer Research Fund Colorectal Cancer Unit, St Mark's Hospital, Northwick Park, Watford Road, Harrow, Middlesex, HA1 3UJ, UK; Cambridge University Department of Medical Genetics, Molecular Genetics Laboratory, PO Box 158, Addenbrooke's Hospital, Cambridge, CB2 2QQ, UK

**Keywords:** desmoids, familial adenomatous polyposis, clonality, neoplasm, X-chromosome inactivation, HUMARA

## Abstract

Desmoids are poorly-understood, locally aggressive, non-metastasizing fibromatoses that occur with disproportionate frequency in patients with familial adenomatous polyposis (FAP). Their nature is controversial with arguments for and against a neoplastic origin. Neoplastic proliferations are by definition monoclonal, whereas reactive processes originate from a polyclonal background. We examined clonality of 25 samples of desmoid tissue from 11 female FAP patients by assessing patterns of X-chromosome inactivation to calculate a clonality ratio. Polymerase chain reaction (PCR) amplification of a polymorphic CAG short tandem repeat (STR) sequence adjacent to a methylation-sensitive restriction enzyme site within the human androgen receptor (HUMARA) gene using fluorescent-labelled primers enabled analysis of PCR products by Applied Biosystems Genescan II^TM^software. Twenty-one samples from nine patients were informative for the assay. Samples from all informative cases comprised a median of 66% (range 0–75%) clonal cells but from the six patients with a clonality ratio ≤0.5 comprised a median of 71% (65–75%) clonal cells. FAP-associated desmoid tumours are true neoplasms. This may have implications in the development of improved treatment protocols for patients with these aggressive tumours. © 2000 Cancer Research Campaign


*British Journal of Cancer* (2000) **82**(4), 827–832
© 2000 Cancer Research Campaign
DOI: 10.1054/ bjoc.1999.1007, available online at http://www.idealibrary.com on IDEAL®

### Desmoids in familial adenomatous polyposis are monoclonal proliferations

**SB Middleton[1], IM Frayling[2] and RKS Phillips[1]**

[1]The Polyposis Registry, Imperial Cancer Research Fund Colorectal Cancer Unit, St Mark's Hospital, Northwick Park, Watford Road, Harrow, Middlesex HA1 3UJ, UK; [2]Cambridge University Department of Medical Genetics, Molecular Genetics Laboratory, PO Box 158, Addenbrooke's Hospital, Cambridge CB2 2QQ, UK

**Summary** Desmoids are poorly-understood, locally aggressive, non-metastasizing fibromatoses that occur with disproportionate frequency in patients with familial adenomatous polyposis (FAP). Their nature is controversial with arguments for and against a neoplastic origin. Neoplastic proliferations are by definition monoclonal, whereas reactive processes originate from a polyclonal background. We examined clonality of 25 samples of desmoid tissue from 11 female FAP patients by assessing patterns of X-chromosome inactivation to calculate a clonality ratio. Polymerase chain reaction (PCR) amplification of a polymorphic CAG short tandem repeat (STR) sequence adjacent to a methylation-sensitive restriction enzyme site within the human androgen receptor (HUMARA) gene using fluorescent-labelled primers enabled analysis of PCR products by Applied Biosystems Genescan II™ software. Twenty-one samples from nine patients were informative for the assay. Samples from all informative cases comprised a median of 66% (range 0–75%) clonal cells but from the six patients with a clonality ratio ≤0.5 comprised a median of 71% (65–75%) clonal cells. FAP-associated desmoid tumours are true neoplasms. This may have implications in the development of improved treatment protocols for patients with these aggressive tumours. © 2000 Cancer Research Campaign

**Keywords**: desmoids; familial adenomatous polyposis; clonality; neoplasm; X-chromosome inactivation; HUMARA

Desmoids are locally aggressive proliferations of fibroblasts and are classified as fibromatoses (Stout, 1951), a group including Dupuytren's contracture, plantar fibromatosis, Peyronie's disease, hypertrophic scars and low-grade fibrosarcomas. The incidence of desmoids is only 2–4 per million per year (Reitamo et al, 1982), but 2% of all desmoid tumours are associated with familial adenomatous polyposis (FAP) and the risk to an FAP patient is 1000 times that of the general population (Gurbuz et al, 1994). Approximately 10% of FAP patients will develop a desmoid (Church, 1994), 70% of which will be intra-abdominal (Gurbuz et al, 1994) where they lead to major morbidity and mortality usually as a result of small bowel obstruction and ischaemia, ureteric obstruction or chronic sepsis.

The nature of desmoids remains controversial. Their locally aggressive behaviour and potentially rapid progression (Church, 1995) suggests a neoplastic origin. Furthermore, intra-abdominal desmoids removed surgically recur in as many as 88% of cases (Lotfi et al, 1989) and there is evidence of response to chemotherapy. On the other hand they never metastasize and there is only one report of frankly malignant change in a desmoid (Soule and Scanlon, 1962). In addition, almost 50% do not progress and 10% regress spontaneously (Church, 1995). Histologically they resemble hypertrophic scars, consisting of mature fibroblasts (Hunt et al, 1960) or myofibroblasts (Goellner and Soule, 1980) with few mitoses and no atypia. Finally, they do not express telomerase (Scates et al, 1998) and these features are suggestive of a non-neoplastic process.

*Received 30 June 1999*
*Revised 4 October 1999*
*Accepted 6 October 1999*

*Correspondence to:* RKS Phillips

There is specific evidence favouring a neoplastic origin for both sporadic desmoids, which are associated with trisomies 8 (Dal Cin et al, 1994) and 20 (Dal Cin et al, 1995) and FAP-associated desmoids, where somatic mutations as well as the *APC* germline mutation have been identified (Miyaki et al, 1993; Palmirotta et al, 1995). This is consistent with the two-hit loss of suppressor gene hypothesis and is strong evidence that desmoids are indeed true neoplasms.

The fundamental property of a neoplastic proliferation which differentiates it from a reactive process is that it originates from a single cell that acquires a selective growth advantage (Knudson, 1985). A neoplastic proliferation is therefore by definition monoclonal. At the late blastocyst stage of embryogenesis one or other of the paternally or maternally-derived X-chromosomes is randomly inactivated in each cell; that pattern is reproduced in each daughter cell (Lyon, 1972) and is reliably transmitted during malignant transformation (Wainscoat and Fey, 1990). Monoclonal proliferations therefore display a non-random pattern of X-chromosome inactivation, whereas polyclonal processes display a random pattern of X-chromosome inactivation (Vogelstein et al, 1987). Initial techniques to assess clonality based on X-chromosome inactivation involved isoenzymes of glucose-6-phosphate dehydrogenase (GAPDH) (Fialkow et al, 1967) but these were inefficient and have now been replaced by a reliable polymerase chain reaction (PCR)-based assay (Allen et al, 1992; Willman et al, 1994).

Patterns of X-chromosome inactivation can be assessed at an X-linked locus if that locus can be identified separately on each X-chromosome and if it is possible to distinguish between the active and inactivated X-chromosome. The human androgen receptor gene (HUMARA) at Xcen-q13 fulfils these criteria. It contains a polymorphic hypervariable short tandem repeat (STR)

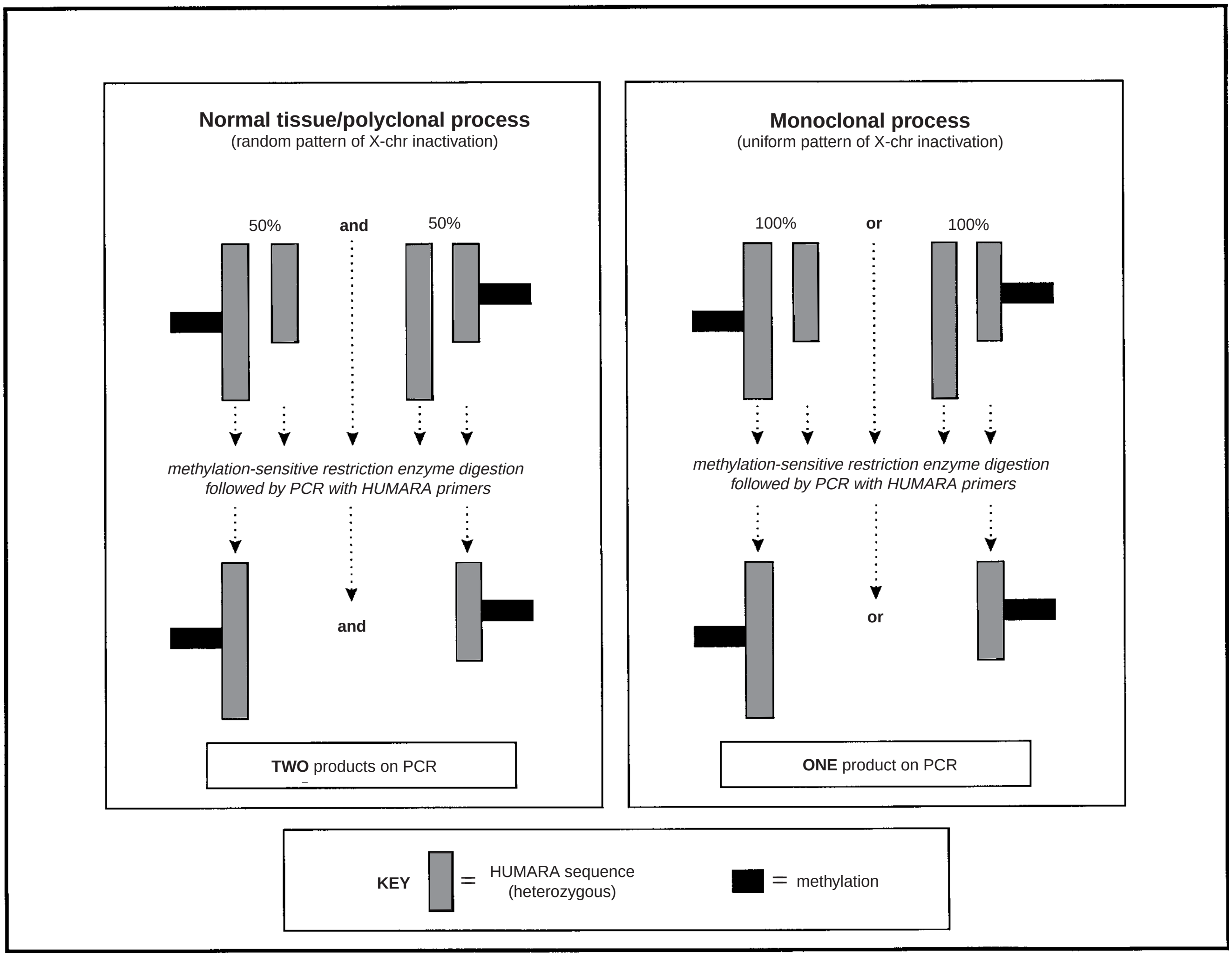

**Figure 1:** Schematic diagram of the HUMARA PCR-based assay sequence ($(CAG)_n$ where $n$ = 11–31) and heterozygosity is greater than 90% (Allen et al, 1992) as a result of the paternal and maternal origins of each X-chromosome. Immediately upstream of the CAG repeat there is a methylation-sensitive *HpaII* and *HhaI* restriction enzyme site which is methylated in association with X-chromosome inactivation (Allen et al, 1992), preventing digestion by either *HpaII* or *HhaI*. PCR amplification using oligonucleotide primers directed towards HUMARA produces two products, corresponding to one HUMARA sequence on each X-chromosome. PCR carried out following *HpaII*/*HhaI* digestion leads to two products if there is a random pattern of inactivation but only one product if there is a non-random pattern (Figure 1). By these means it is possible to distinguish between monoclonal and polyclonal proliferations. The technique has been used to demonstrate that sporadic desmoids are clonal in origin (Li et al, 1996; Alman et al, 1997; Lucas et al, 1997) but there are important differences between sporadic and FAP-associated desmoids, where there is a less obvious female preponderance (Gurbuz et al, 1994; Rodriguez-Bigas et al, 1994). In addition, sporadic desmoids occur in intra-abdominal sites in less than 10% of cases (Reitamo et al, 1986) and while most FAP-associated desmoids follow trauma (Lotfi et al, 1989; Rodriguez-Bigas et al, 1994), only about 30% of sporadic desmoids do so (Hayry et al, 1982). Finally, these studies have used radiolabelled primers and phosphoimaging but the use of fluorescent-labelled primers and commercial software analysis avoids radioactive materials and enables more objective analysis (Delabesse et al, 1995). We used this modification to examine patterns of X-chromosome inactivation at the HUMARA locus in FAP-associated desmoids.

### MATERIALS AND METHODS

### Tissues

Desmoid tumours and control blood samples were obtained from female FAP patients undergoing surgery at St Mark's Hospital. Clinical data were obtained from the St Mark's Polyposis Registry. Colorectal cancer tissue samples were obtained as positive controls. All tissue specimens were confirmed histologically.

### DNA extraction and restriction enzyme digestion

DNA was obtained by proteinase K digestion, phenol–chloroform extraction and ethanol precipitation. One microgram of each DNA sample was incubated for 16 h at 37°C with 10 IU of restriction enzyme (*HhaI*, Amersham Pharmacia, UK; *HpaII*, Promega,

© 2000 Cancer Research Campaign

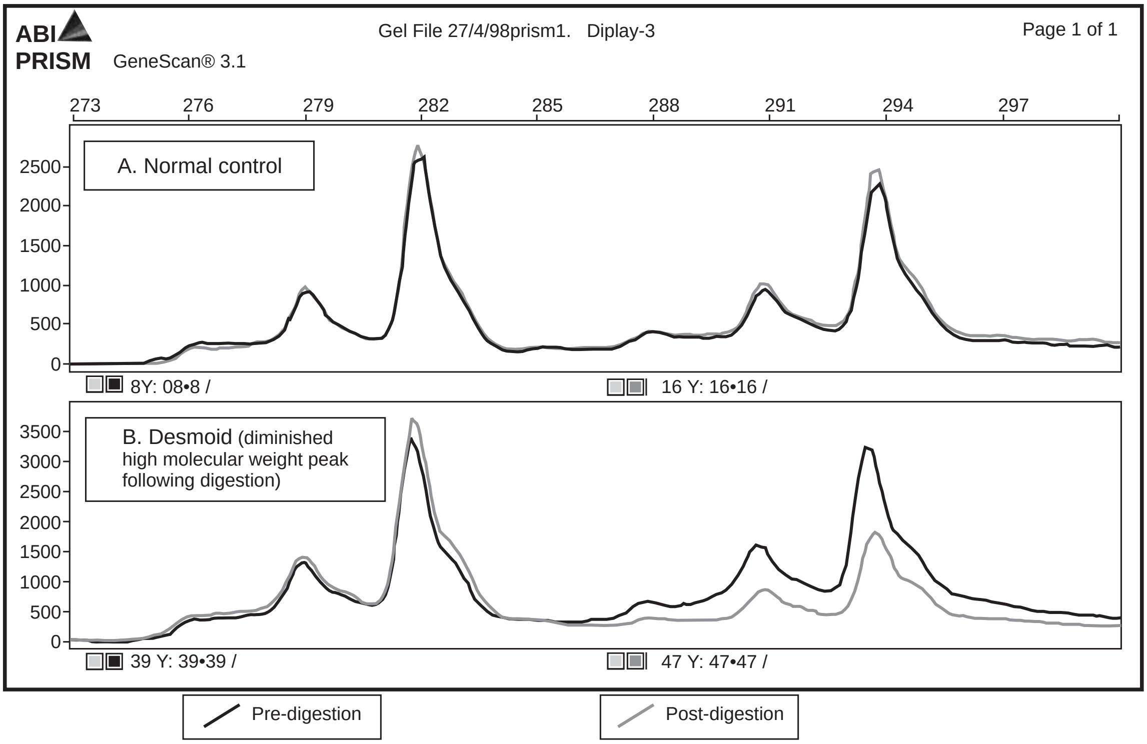

**Figure 2:** Genescan™ analysis of desmoid tumour and normal control

USA) in a final volume of 20 μl according to the manufacturers' recommendations. In each case a control sample was incubated in a similar volume without restriction enzyme.

### PCR amplification

A 20 μl PCR was carried out using 1 μl of each incubated DNA sample in 12.9 μl water, 2 μl of 2.5 μM fluorescent-labelled oligonucleotide HUMARA primers (5′-HEX-GCTGTGAAG-GTTGCTGTTCCTCAT-3′ and 5′-TCCAGAATCTGTTCCA-GAGCGTGC-3′), 1.6 μl dNTPs, 0.4 μl 7-deaza-2′-deoxy GTP (Boehringher Mannheim), 2 μl reaction buffer (500 mM; potassium chloride (KCl); 100 mM Tris–HCl, pH 9; 1% Triton® × 100; 25 mM magnesium chloride ($MgCl_2$), and 0.1 μl *Taq* DNA polymerase. The primers were provided by Imperial Cancer Research Fund's laboratories. Initial denaturation was carried out in a preheated thermal cycler at 95°C for 5 min, followed by 35 cycles of 94°C for 1 min, 55°C for 1 min and 72°C for 1 min. Final extension continued for a further 5 min. Combination with loading buffer and gel electrophoresis through 2% agarose confirmed PCR products and following migration using a 10% denaturing polyacrylamide gel on an automatic Applied Biosystems ABI 377 sequence analyser, fluorescent peaks were identified using the Genescan II™ software. All assays were repeated in triplicate.

### Interpretation

Superimposing Genescan™ peaks for individual samples before and following restriction enzyme digestion gave an immediate impression of clonality (Figure 2) but an inactivation or clonality ratio (CR) was calculated for each according to a previously described technique (Willman et al, 1994). In this way the amplifi-

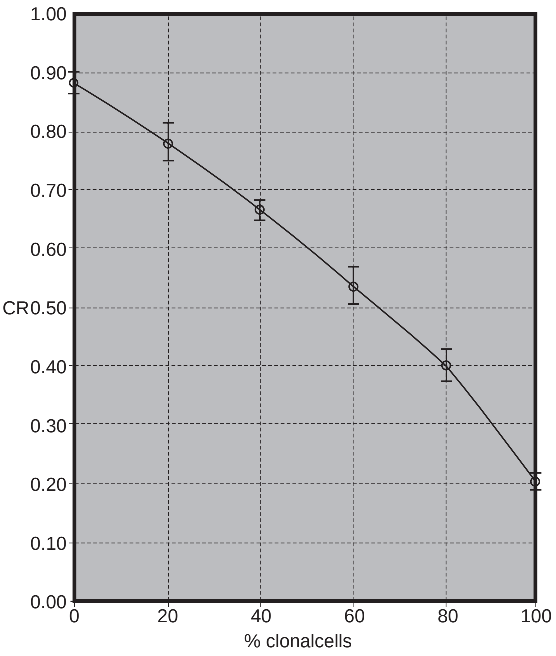

**Graph data**

| % clonal cells | CR | | | | mean | sem | sd |
|---|---|---|---|---|---|---|---|
| | 1 | 2 | 3 | | | | |
| **0** | 0.853 | 0.914 | 0.887 | | **0.88** | 0.018 | 0.031 |
| **20** | 0.774 | 0.841 | 0.732 | | **0.78** | 0.032 | 0.055 |
| **40** | 0.648 | 0.700 | 0.651 | | **0.67** | 0.017 | 0.029 |
| **60** | 0.495 | 0.598 | 0.514 | | **0.54** | 0.032 | 0.055 |
| **80** | 0.378 | 0.448 | 0.355 | | **0.39** | 0.028 | 0.048 |
| **100** | 0.225 | 0.197 | 0.175 | | **0.20** | 0.014 | 0.025 |

**Figure 3:** Standard curve of clonality ratio (CR) v. percentage clonal cells for control samples cation ratio (AR) for a sample is $P_{HMW}/P_{LMW}$ where $P_{HMW}$ is the PCR product of the high molecular weight product and $P_{LMW}$ is the PCR product of the low molecular weight product. $P_{HMW}/P_{LMW}$ is calculated as $[A_{stut(HMW)} + A_{prim(HMW)}]/[A_{stut(LMW)} + A_{prim(LMW)}]$ where $A_{prim}$ is the area (or height) of the primary peak and $A_{stut}$ is the area (or height) of the highest molecular weight stutter peak on the Genescan™ image of that sample. The CR is $AR_{undig}/AR_{dig}$ where $AR_{undig}$ is the amplification ratio of undigested tumour DNA and $AR_{dig}$ is the amplification ratio of digested tumour DNA. If the CR is greater than 1 the reciprocal figure is used. The CR provides an objective assessment of the degree to which the lower or higher molecular weight PCR product is absent following restriction enzyme digestion and therefore the degree to which the sample is clonal. Clonality ratios between 1 and 0 are obtained where one indicates a polyclonal origin and zero indicates a monoclonal origin for the sample. To allow for unequal lyonization (Fey et al, 1992), the CR is divided by the CR calculated in a similar fashion for the corresponding blood control (El Kassar et al, 1998). A final, corrected CR ≤ 0.5 has been considered indicative of a clonal composition (Li et al, 1996). To improve objectivity, we calculated CRs for samples of proportionally mixed colorectal cancer DNA (considered to be 100% clonal) and blood lymphocyte DNA (considered to be 0% clonal). It was then possible to construct a standard curve of CR against percentage clonal cells (Figure 3) such that the proportion of clonal cells in each desmoid sample could be deduced from the curve when the CR was known for that sample.

### RESULTS (table 1)

Sixteen intra-abdominal and nine abdominal wall samples of histologically confirmed desmoid tissue were available from 11 female FAP patients. Control blood samples were available for seven of the patients, whose ages ranged from 16 to 45 years (median 28 years). Two patients had been treated with non-steroidal anti-inflammatory medication and two with toremifene, an anti-oestrogen. One had been treated with chemotherapy 3 years earlier but none had received radiotherapy. The HUMARA sequence was informative in 12 intra-abdominal and nine abdominal wall samples from nine of the 11 patients. All control blood samples were informative, although two related to non-informative desmoid samples. Informative results were achieved more frequently with *HhaI* than *HpaII*, but in those samples which were informative with both enzymes, very similar CRs were obtained with each. Multiple samples from individual patients produced similar results apart from samples 8, 9 and 17 (Table 1), which yielded unusually high CRs compared to other samples from the same patient. Clonality ratios calculated from peak areas or peak heights yielded almost identical results. As expected, all primary peaks observed were multiples of 3 base pairs apart due to the tri-nucleotide repeat within HUMARA (Figure 2). Samples from six patients were found to have a final CR ≤ 0.5 and those from a further three had a CR > 0.5. The corresponding percentage of clonal cells calculated from standard curves ranged from 0% to 75% (median 66%) for all patients or 65% to 75% (median 71%) for those with a CR ≤ 0.5. Clonality ratios for the control blood samples are shown in Table 1. The mean figure of 0.76 was used to correct for unequal lyonization in patients for whom a control sample was unavailable. There was no obvious relationship between clonality and either germline mutation, somatic mutation or previous treatment, although it is of interest to note that patient 7 had received chemotherapy and her desmoid samples had a low proportion of clonal cells.

**Table 1** Clonality ratios and clonal cell proportions

| Patient | Age | Sample | Site | Germline mutation[a] | Somatic mutation[a] | CR | Control CR | Controlled CR | Final CR | % clonal cells |
|---|---|---|---|---|---|---|---|---|---|---|
| 1 | 42 | 1 | Intra-abdominal | 1392 | ND | NI | 0.74 | NI | NI | NI |
| | | 2 | Intra-abdominal | | | NI | 0.74 | NI | NI | NI |
| 2 | 25 | 3 | Intra-abdominal | 1084 | 1542 | 0.37 | 0.85 | 0.44 | **0.44** | **73** |
| 3 | 24 | 4 | Intra-abdominal | ND | ND | 0.50 | **0.76** | 0.66 | **0.66** | **42** |
| 4 | 40 | 5 | Abdominal wall | ND | 1433 | 0.32 | 0.73 | 0.44 | **0.50**[b] | **65** |
| | | 6 | Abdominal wall | | | 0.40 | 0.73 | 0.55 | | |
| | | 7 | Abdominal wall | | | 0.36 | 0.73 | 0.49 | | |
| | | 8 | Abdominal wall | | | 0.52 | 0.73 | 0.71 | | |
| | | 9 | Abdominal wall | | | 0.61 | 0.73 | 0.84 | | |
| | | 10 | Abdominal wall | | | 0.20 | 0.73 | 0.27 | | |
| | | 11 | Abdominal wall | | | 0.18 | 0.73 | 0.25 | | |
| | | 12 | Abdominal wall | | | 0.35 | 0.73 | 0.48 | | |
| 5 | 45 | 13 | Intra-abdominal | ND | ND | NI | 0.78 | NI | NI | NI |
| | | 14 | Intra-abdominal | | | NI | 0.78 | NI | NI | NI |
| 6 | 31 | 15 | Intra-abdominal | 1461 | 1483 | 0.33 | 0.67 | 0.49 | **0.49** | **66** |
| 7 | 28 | 16 | Intra-abdominal | 1450 | ND | 0.63 | 0.84 | 0.75 | **0.72**[b] | **32** |
| | | 17 | Intra-abdominal | | | 0.75 | 0.84 | 0.89 | | |
| | | 18 | Intra-abdominal | | | 0.57 | 0.84 | 0.68 | | |
| | | 19 | Intra-abdominal | | | 0.50 | 0.84 | 0.60 | | |
| | | 20 | Intra-abdominal | | | 0.60 | 0.84 | 0.71 | | |
| | | 21 | Intra-abdominal | | | 0.60 | 0.84 | 0.71 | | |
| 8 | 20 | 22 | Intra-abdominal | 1397 | 1543 | 0.36 | **0.76** | 0.47 | **0.47** | **69** |
| 9 | 45 | 23 | Intra-abdominal | ND | ND | 0.32 | **0.76** | 0.42 | **0.42** | **75** |
| 10 | 16 | 24 | Abdominal wall | ND | 1462 | 0.71 | **0.76** | 0.93 | **0.93** | **0** |
| 11 | 22 | 25 | Intra-abdominal | 233 | ND | 0.30 | 0.71 | 0.42 | **0.42** | **75** |

CR = clonality ratio; **control CR** = control not available (Mean of available control CRs used); NI = non-informative; ND = not detected. [a]*APC* mutation detected in previous work; [b]Mean CR for multiple samples.

*© 2000 Cancer Research Campaign*

### DISCUSSION

As screening for FAP becomes more efficient and surgery more sophisticated, the relative importance of extracolonic manifestations of FAP is increasing (Belchetz et al, 1996). In addition, the combination of the location and unpredictable nature of FAP-associated desmoids means that they are one of the two leading causes of death in FAP patients who have undergone prophylactic colectomy (Nugent et al, 1993). Desmoids, however, remain poorly understood and as a result treatment is poor and mortality high (Rodriguez-Bigas et al, 1994). Currently the commonest forms of treatment for desmoids are sulindac, tamoxifen or toremifene (Brooks et al, 1992; Tsukada et al, 1992), radiotherapy (Kiel and Suit, 1984), cytotoxic chemotherapy (Lynch et al, 1994; Hamilton et al, 1996) or excisional surgery. All of these modalities have limited efficacy and potential problems. An understanding of the nature of desmoids will encourage the development of more appropriate tailored treatment protocols.

Assessment of clonality by the examination of patterns of X-chromosome inactivation has proved to be a reliable technique and has demonstrated that FAP-associated desmoid in our series are true neoplasms. Although the number of patients involved was small, most other authors using a similar technique have used even fewer. This can be explained by the low incidence of desmoids and an increasing tendency to avoid surgery for FAP-related desmoid disease. The only limitations of the HUMARA PCR-based assay are a requirement for female tissue samples and heterozygous HUMARA sequences. As reported by others (Li et al, 1996) we found the addition of 7-deaza-2′-deoxy GTP to the dNTP mixture improved the PCR process significantly. This manoeuvre is said to reduce the formation of DNA secondary structures when carrying out PCR assays whose products contain CpG-rich regions (Mutter and Boynton, 1995).

The technique enables samples to be easily and rapidly recognized as informative or non-informative and an idea of the result may be obtained by simple observation of the Genescan™ images. Other authors using radiolabelled primers and phosphoimaging have relied on visual assessment alone to designate clonality (Alman et al, 1997). The use of fluorescent-labelled primers not only avoids the safety and environmental issues of radioactive material but allows a quantitative analysis of clonality through the calculation of a CR. The only disadvantage is the difficulty in relating the CR to the true clonality of a sample. Clonality ratios of 1 do not occur as there is frequently unequal lyonization of normal tissues. Clonality ratios of 0 do not occur as there is contamination of tumour specimens with other non-clonal cells such as lymphocytes, peritoneal cells and vascular elements. Microdissection techniques have enabled lower CRs to be obtained with other tumour specimens, including sporadic desmoids (Lucas et al, 1997), but we have found the technique to be difficult and unhelpful with FAP-associated desmoids due to their often sparse cellularity.

A positive clonality result is indicated by a significant monoclonal contamination of a polyclonal background and it has been shown that the percentage of clonal cells can be estimated with an error of ± 10% and that a clonal population of cells can be detected if they represent greater than 10–15% of cells in a polyclonal background (Delabesse et al, 1997). All of our samples comprised a greater proportion of clonal cells than this minimum. The use of blood lymphocytes as control tissue for distinguishing between skewed inactivation and clonal proliferation has been shown to be reliable in young females (Tonon et al, 1998); the median age in our series was 28 years.

The confirmation that FAP-associated desmoids are neoplastic improves understanding of their development. Fibroblasts in patients with FAP are known to be abnormal (Pfeffer et al, 1976). White plaques or strands known as desmoid precursor lesions (DPLs) develop from fibroblasts in the mesentery of a third of post-operative patients with FAP and mesenteric fibromatosis (tethering or puckering of the mesentery not amounting to a mass but detectable on computerized tomography scanning) occurs in 20–25% of cases (Clark et al, 1998). Finally around 10% develop a desmoid. Our results indicate that FAP-associated desmoids are true neoplasms which may fit into a cascade analogous to the multi-step process in cancer development where separate clonal expansions follow individual mutations (Vogelstein and Kinzler, 1993). It may be that fibroblasts in FAP containing an *APC* germline mutation are at risk of forming a DPL, although this mechanism is unclear and it has been shown that transient transfection of wild-type *APC* into desmoid cell cultures actually decreases proliferation (Li et al, 1998). During the peri- or post-operative period, a fibroblast within the DPL may acquire a somatic mutation and develop a selective growth advantage, enabling clonal expansion to form a true desmoid.

This study has addressed the question of the nature of FAP-associated desmoids. Whether it will allow rationalization of treatment protocols for these locally aggressive tumours which have high mortality in a young population, remains to be seen. It suggests the need for further investigation into the role of antisarcoma chemotherapy, a potentially lengthy and toxic regime whose efficacy is as yet unclear. Of interest, desmoid samples from the one patient in this study who had previously received chemotherapy had a low clonality ratio, but our (unpublished) experience of chemotherapy is disappointing and we are not yet convinced of its role in the treatment of desmoids. Finally, further work is required to ascertain the nature of the somatic mutation occurring in the perioperative period which precipitates clonal expansion to form an FAP-associated desmoid.

### ACKNOWLEDGEMENTS

The authors wish to thank Ms SK Clark MD FRCS who provided details of the *APC* mutations listed in Table 1.

### REFERENCES

Allen RC, Zoghbi HY, Moseley AB, Rosenblatt HM and Belmont JW (1992) Methylation of *HpaII* and *HhaI* sites near the polymorphic CAG repeat in the human androgen-receptor gene corresponds with X-chromosome inactivation. *Am J Hum Genet* **51**: 1229–1239

Alman BA, Pajerski ME, Diaz-Cano S, Corboy K and Wolfe HJ (1997) Aggressive fibromatosis (desmoid tumour) is a monoclonal disorder. *Diagn Mol Pathol* **6**: 98–101

Belchetz LA, Berk T, Bapat B, Cohen Z and Gallinger S (1996) Changing causes of mortality in patients with FAP. *Dis Colon Rectum* **39**: 384–387

Brooks MD, Ebbs SR, Colletta AA and Baum M (1992) Desmoid tumours treated with triphenylethylenes. *Eur J Cancer* **28A**: 1014–1018

Church JM (1994) Desmoid tumours in familial adenomatous polyposis. *Surg Oncol Clin N Am* **3**: 435–447

Church JM (1995) Desmoid tumours in patients with familial adenomatous polyposis. *Semin Colon Rectal Surg* **6**: 29–32

© 2000 Cancer Research Campaign 

Clark SK, Johnson Smith TGP, Katz DE, Reznek RH and Phillips RKS (1998) Identification and progression of a desmoid precursor lesion in patients with familial adenomatous polyposis. *Br J Surg* **85**: 970–973

Dal Cin P, Sciot R, Aly MS, Delabie J, Stas M, De Wever I, Van Damme B and Van den Berghe H (1994) Some desmoid tumors are characterized by trisomy 8. *Genes Chromosomes Cancer* **10**: 131–135

Dal Cin P, Sciot R, Van Damme B, De Wever I and Van den Berghe H (1995) Trisomy 20 characterizes a second group of desmoid tumors. *Cancer Genet Cytogenet* **79**: 189

Delabesse E, Aral S, Kamoun P, Varet B and Turhan AG (1995) Quantitative non-radioactive clonality analysis of human leukemic cells and progenitors using the human androgen receptor (AR) gene. *Leukemia* **9**: 1578–1582

Delabesse E, Oksenhendler E, Lebbe C, Verola O, Varet B and Turhan AG (1997) Molecular analysis of clonality in Kaposi's sarcoma. *J Clin Pathol* **50**: 664–668

El Kassar N, Hetet G, Briere J and Grandchamp B (1998) X-chromosome inactivation in healthy females: incidence of excessive lyonisation with age and comparison of assays involving DNA methylation and transcript polymorphisms. *Clin Chem* **44**: 61–67

Fey MF, Peter HJ, Hinds HL, Zimmermann A, Liechti-Gallati S, Gerber H, Studer H and Tobler A (1992) Clonal analysis of human tumors with M27 beta, a highly informative polymorphic X chromosomal probe. *J Clin Invest* **89**: 1438–1444

Fialkow PJ, Gartler SM and Yoshida A (1967) Clonal origin of chronic myelocytic leukaemia in man. *Proc Natl Acad Sci USA* **58**: 1468–1471

Goellner ER and Soule EH (1980) Desmoid tumours. An ultrastructural study of eight cases. *Hum Pathol* **11**: 43–50

Gurbuz AK, Giardiello FM, Petersen GM, Krush AJ, Offerhaus GJ, Booker SV, Kerr MC and Hamilton SR (1994) Desmoid tumours in familial adenomatous polyposis. *Gut* **35**: 377–381

Hamilton L, Blackstein M, Berk T, McLeod RS, Gallinger S, Madlensky L and Cohen Z (1996) Chemotherapy for desmoid tumours in association with familial adenomatous polyposis: a report of three cases. *Can J Surg* **39**: 247–252

Hayry P, Reitamo JJ, Totterman S, Hopfner-Hallikainen D and Sivula A (1982) The desmoid tumor. II. Analysis of factors possibly contributing to the etiology and growth behavior. *Am J Clin Pathol* **77**: 674–680

Hunt RTN, Morgan HC and Ackerman LV (1960) Principles in the management of extra-abdominal desmoids. *Cancer* **13**: 825

Kiel KD and Suit HD (1984) Radiation therapy in the treatment of aggressive fibromatoses (desmoid tumors). *Cancer* **54**: 2051–2055

Knudson AGJ (1985) Hereditary cancer, oncogenes and anti-oncogenes. *Cancer Res* **45**: 1437–1443

Li C, Bapat B and Alman BA (1998) Adenomatous polyposis coli gene mutation alters proliferation through its beta-catenin-regulatory function in aggressive fibromatosis (desmoid tumour). *Am J Pathol* **153**: 709–714

Li M, Cordon-Cardo C, Gerald WL and Rosai J (1996) Desmoid fibromatosis is a clonal process. *Hum Pathol* **27**: 939–943

Lotfi AM, Dozois RR, Gordon H, Hruska LS, Weiland LH, Carryer PW and Hurt RD (1989) Mesenteric fibromatosis complicating familial adenomatous polyposis: predisposing factors and results of treatment. *Int J Colorectal Dis* **4**: 30–36

Lucas DR, Shroyer KR, McCarthy PJ, Markham NE, Fujita M and Enomoto TE (1997) Desmoid tumor is a clonal cellular proliferation: PCR amplification of HUMARA for analysis of patterns of X-chromosome inactivation. *Am J Surg Pathol* **21**: 306–311

Lynch HT, Fitzgibbons R, Jr, Chong S, Cavalieri J, Lynch J, Wallace F and Patel S (1994) Use of doxorubicin and dacarbazine for the management of unresectable intra-abdominal desmoid tumors in Gardner's syndrome. *Dis Colon Rectum* **37**: 260–267

Lyon MF (1972) X-chromosome inactivation and developmental patterns in mammals. *Biol Rev Camb Philos Soc* **47**: 1–35

Miyaki M, Konishi M, Kikuchi-Yanoshita R, Enomoto M, Tanaka K, Takahashi H, Muraoka M, Mori T, Konishi F and Iwama T (1993) Coexistence of somatic and germ-line mutations of *APC* gene in desmoid tumors from patients with familial adenomatous polyposis. *Cancer Res* **53**: 5079–5082

Mutter GL and Boynton KA (1995) PCR bias in amplification of androgen receptor alleles, a trinucleotide repeat marker used in clonality studies. *Nucleic Acids Res* **23**: 1411–1418

Nugent KP, Spigelman AD and Phillips RKS (1993) Life expectancy after colectomy and ileorectal anastomosis for familial adenomatous polyposis. *Dis Colon Rectum* **36**: 1059–1062

Palmirotta R, Curia MC, Esposito DL, Valanzano R, Messerini L, Ficari F, Brandi ML, Tonelli F, Mariani-Costantini R and Battista P (1995) Novel mutations and inactivation of both alleles of the *APC* gene in desmoid tumors. *Hum Mol Genet* **4**: 1979–1981

Pfeffer L, Lipkin M, Stutman O and Kopelovich L (1976) Growth abnormalities of cultured human skin fibroblasts derived from individuals with hereditary adenomatosis of the colon and rectum. *J Cell Physiol* **89**: 29–37

Reitamo JJ, Hayry P, Nykyri E and Saxen E (1982) The desmoid tumor. I. Incidence, sex-, age- and anatomical distribution in the Finnish population. *Am J Clin Pathol* **77**: 665–673

Reitamo JJ, Scheinin TM and Hayry P (1986) The desmoid syndrome. New aspects in the cause, pathogenesis and treatment of the desmoid tumor. *Am J Surg* **151**: 230–237

Rodriguez-Bigas MA, Mahoney MC, Karakousis CP and Petrelli NJ (1994) Desmoid tumors in patients with familial adenomatous polyposis. *Cancer* **74**: 1270–1274

Scates DK, Clark SK, Phillips RKS and Venitt S (1998) Lack of telomerase in desmoids occurring sporadically and in association with familial adenomatous polyposis. *Br J Surg* **85**: 965–969

Soule EH and Scanlon PW (1962) Fibrosarcoma arising in an extra-abdominal desmoid; report of a case. *Mayo Clin Proc* **37**: 433–451

Stout AP (1951) The fibromatoses and fibrosarcoma. *Bull Hospital Joint Dis* **12**: 126–130

Tonon L, Bergamaschi G, Dellavecchia C, Rosti V, Lucotti C, Malabarba L, Novella A, Vercesi E, Frassoni F and Cazzola M (1998) Unbalanced X-chromosome inactivation in haemopoietic cells from normal women. *Br J Haematol* **102**: 996–1003

Tsukada K, Church JM, Jagelman DG, Fazio VW, McGannon E, George CR, Schroeder T, Lavery I and Oakley J (1992) Noncytotoxic drug therapy for intra-abdominal desmoid tumor in patients with familial adenomatous polyposis. *Dis Colon Rectum* **35**: 29–33

Vogelstein B and Kinzler K (1993) The multistep nature of cancer. *Trends Genet* **9**: 138

Vogelstein B, Fearon ER, Hamilton SR, Preisinger AC, Willard HF, Michelson AM, Riggs AD and Orkin SH (1987) Clonal analysis using recombinant DNA probes from the X-chromosome. *Cancer Res* **47**: 4806–4813

Wainscoat JS and Fey MF (1990) Assessment of clonality in human tumors: a review. *Cancer Res* **50**: 1355–1360

Willman CL, Busque L, Griffith BB, Favara BE, McClain KL, Duncan MH and Gilliland DG (1994) Langerhans'-cell histiocytosis (Histiocytosis X) – a clonal proliferative disease. *N Engl J Med* **331**: 154–160

*© 2000 Cancer Research Campaign*
